# Analysis of Asymptomatic and Presymptomatic Transmission in SARS-CoV-2 Outbreak, Germany, 2020

**DOI:** 10.3201/eid2704.204576

**Published:** 2021-04

**Authors:** Jennifer K. Bender, Michael Brandl, Michael Höhle, Udo Buchholz, Nadine Zeitlmann

**Affiliations:** Robert Koch Institute, Wernigerode, Germany (J.K. Bender);; European Centre for Disease Prevention and Control, Stockholm, Sweden (J.K. Bender, M. Brandl);; Robert Koch Institute, Berlin, Germany (M. Brandl, M. Höhle, U. Buchholz, N. Zeitlmann);; Stockholm University, Stockholm (M. Höhle);; Federal Institute for Quality Assurance and Transparency in Healthcare, Berlin (M. Höhle)

**Keywords:** COVID-19, SARS-CoV-2, severe acute respiratory syndrome coronavirus 2, viruses, respiratory infections, zoonoses, coronavirus disease, risk, disease transmission, infectious, asymptomatic diseases, epidemiology, contact, Germany

## Abstract

We determined secondary attack rates (SAR) among close contacts of 59 asymptomatic and symptomatic coronavirus disease case-patients by presymptomatic and symptomatic exposure. We observed no transmission from asymptomatic case-patients and highest SAR through presymptomatic exposure. Rapid quarantine of close contacts with or without symptoms is needed to prevent presymptomatic transmission.

During the ongoing coronavirus disease (COVID-19) pandemic, worldwide, >85 million severe acute respiratory syndrome coronavirus 2 (SARS-CoV-2) infections had been reported as of January 7, 2021 (https://covid19.who.int). Although it was clear from the beginning of the pandemic that symptomatic transmission of SARS-CoV-2 occurs, presymptomatic transmission has also been described (*1**–**6*). Furthermore, transmission from asymptomatic cases was deemed possible on the basis of findings that viral load of asymptomatic cases was similar to that of symptomatic cases (*7*). Understanding how transmission occurs from asymptomatic cases and from symptomatic cases in their presymptomatic and symptomatic phase, as well as the frequency of transmission, is essential for public health management. We assessed asymptomatic, presymptomatic, and symptomatic transmission during an outbreak investigation of 59 COVID-19 cases by determining secondary attack rates (SAR) according to the respective exposure periods. In addition, we estimated key parameters such as serial interval and incubation period.

## The Study

On February 29, 2020, a COVID-19 case was notified to the local public health authority (LPHA) of a rural district in southern Germany without previously observed community transmission. During their infectious period, the case-patient had attended several carnival events in the district. The LPHA immediately initiated contact tracing, identifying all close contacts; they were quarantined and tested irrespective of symptoms. By the end of March 2020, a cluster of 59 cases had been identified through successive contact tracing activities.

We interviewed the case-patients of the cluster by phone regarding symptoms developed during SARS-CoV-2 infection; potential source cases or events; and household contacts (HCs) and close nonhousehold or other contacts (OCs) in their infectious period (Appendix). We obtained an empirical distribution of the serial interval from the average over all possible transmission trees of the cluster. We obtained generation time and incubation period by averaging over the estimates as described by Reich et al. (*8*) (Appendix).

To estimate SAR and relative risks (RRs) we conducted a retrospective cohort study, including all HCs and OCs as recalled by the case-patients that met inclusion criteria (Appendix). We calculated pooled SAR of HCs and OCs for 2 outcomes, laboratory confirmation (SAR_lab_) and development of respiratory symptoms (SAR_res_) in the following groups: HCs and OCs of asymptomatic case-patients who never experienced symptoms; HCs and OCs of symptomatic case-patients in which the phase with contact could not be specified by the case-patient or with contact in both phases; OCs of symptomatic case-patients with contact only in the presymptomatic phase; and OCs of symptomatic case-patients with contact only in the symptomatic phase.

We were able to contact 53/59 (90%) case-patients. Three case-patients were children <15 years of age (Table 1). Forty-six (87%) were symptomatic, and 7 (13%) were asymptomatic (Appendix Figure 1). The cluster resulted in 144 possible transmission trees, which span over 5 generations (Figure). No secondary transmission resulted from asymptomatic cases. We determined a median serial interval of 3.0 (IQR 1.0–6.0) days and a median incubation period of 4.3 (IQR 2.5–6.5) days (Appendix Table 1).

**Table 1:** Demographics of coronavirus disease case-patients and their contacts in a district in southern Germany*

Case type	No. (%) asymptomatic	No. (%) symptomatic			Total
		Phase not specified or both†	Presymptomatic phase only	Symptomatic phase only	
Case-patients					
Total	7 (13.2)	46 (86.8)	NA	NA	53 (100)
Female	3 (11.5)	23 (88.5)	NA	NA	26 (100)
Male	4 (14.8)	23 (85.2)	NA	NA	27 (100)
Median age	36 (IQR 6–68)	40 (IQR 29–50)	NA	NA	39.5 (IQR 29–50)‡
Contact persons by type of exposure					
HC	7 (16.7)	35 (83.3)	NA	NA	42 (100)
OC	52 (24.5)	48 (22.6)	81 (38.2)	31 (14.6)	212 (100)

*HC, household contact; IQR, interquartile range; NA, not applicable; OC, nonhousehold or other contact. †The phase in which the contact occurred was not specified, or contact occurred in both phases. ‡Three of 53 cases were children <15 y of age.

**Figure:**
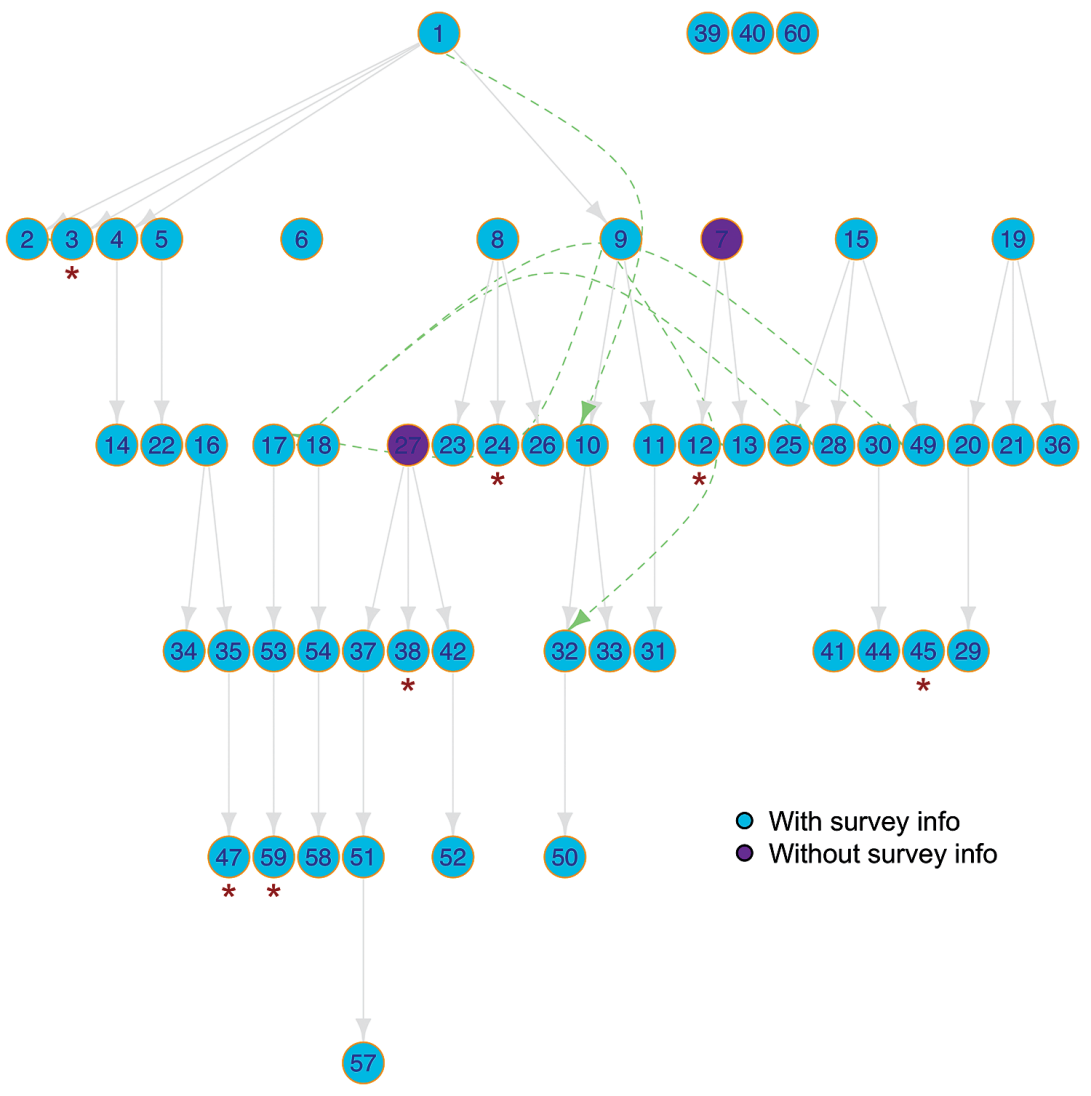
Transmission tree of the investigated cluster of coronavirus disease that evolved in a district in southern Germany. Cases 39, 40, and 60 participated in the survey but were not included in the analysis because we had no information on source case. Cases 7 and 27 did not participate in the survey and thus, no information on source case was available. Dashed lines represent source case–infectee pairs in which the infectee reported >1 possible source case; solid lines represent source case–infectee pairs in which only 1 source case was mapped to the infectee. Asterisks (*) indicate asymptomatic cases. Implausible transmissions (e.g., ID 6) were omitted.

In total, 42 HCs and 212 OCs were included in the cohort study (Table 1). The overall SAR_lab_ was 13% (4/32) for HCs and 14% (20/148) for OCs. The overall SAR_res_ was 29% (12/42) for HCs and 17% (29/170) for OCs (Table 2). We did not identify any HC who tested positive or experienced respiratory symptoms after contact with asymptomatic case-patients. Neither SAR_lab_ nor SAR_res_ of HCs of symptomatic case-patients were significantly higher compared with HCs of asymptomatic cases (SAR_lab_ p = 1.0; SAR_res_ p = 0.23). We observed no laboratory-confirmed SARS-CoV-2 transmission from asymptomatic case-patients to any of the 22 OCs (Table 2; Appendix Figure 2). SAR_lab_ was highest for OCs with contact during the case-patients’ presymptomatic phases (21%; 15/72) yielding a RR of 6.5 (95% CI 1.1–∞) when compared with contacts of asymptomatic case-patients. Adjusting for case-patients’ age, sex, and number of contact persons showed no substantial changes in the magnitude of estimates (data not shown). Presymptomatic transmission accounted for >75% of all transmissions to OCs in the cohort (Appendix).

**Table 2:** Secondary attack rates among contacts of coronavirus disease case-patients in a district in southern Germany*

Clinical symptoms of source case	No. contacts tested positive or experienced respiratory symptoms	Total no. contacts	SAR, %	RR (95% CI)
Household contacts SAR_lab_				
Asymptomatic	0	4	0	Reference
Symptomatic, phase not specified or both†	4	28	14.3	0.8 (0.09–∞)
Total	4	32	12.5	
Household contacts SAR_res_				
Asymptomatic	0	7	0	Reference
Symptomatic, phase not specified or both	12	35	34.3	3.4 (0.56–∞)
Total	12	42	28.6	
Other contacts SAR_lab_				
Asymptomatic cases	0	22	0	Reference
Symptomatic, phase not specified or both	3	25	12.0	3.4 (0.36–∞)
Symptomatic, presymptomatic phase only	15	72	20.8	6.5 (1.1–∞)
Symptomatic, symptomatic phase only	2	29	6.9	1.8 (0.14–∞)
Total	20	148	13.5	
Other contacts SAR_res_				
Asymptomatic cases	2	52	3.8	Reference
Symptomatic, phase not specified or both	4	22	18.2	4.7 (0.68–52)
Symptomatic, presymptomatic phase only	22	67	32.8	8.5 (2.1–75)
Symptomatic, symptomatic phase only	1	29	3.5	0.90 (0.02–17)
Total	29	170	17.1	

*RR relative risk; SAR, secondary attack rate; SAR_lab_ secondary attack rate for laboratory-confirmed SARS-CoV-2–positive contact persons; SAR_res_, secondary attack rate for contact persons who experienced respiratory symptoms after contact.  †The phase in which the contact occurred was not specified, or contact occurred in both phases.

## Conclusions

In this cluster of COVID-19 cases, little to no transmission occurred from asymptomatic case-patients. Presymptomatic transmission was more frequent than symptomatic transmission. The serial interval was short; very short intervals occurred.

The fact that we did not detect any laboratory-confirmed SARS-CoV-2 transmission from asymptomatic case-patients is in line with multiple studies (*9**–**11*). However, Oran et al. have speculated that asymptomatic cases contribute to the rapid progression of the pandemic (*12*). Some studies may be prone to misclassify presymptomatic cases as asymptomatic, leading to heterogeneous reporting of SAR of asymptomatic cases, because of different case definitions or differential duration of follow-up. In our study we used a very sensitive case definition for symptomatic cases that did not require specific symptoms (e.g. fever) to be present. Also, timing of our study would have enabled detection of late onset of symptoms, which gives us confidence in our classification of exposure groups.

The 75% of SARS-CoV-2 transmissions in our cohort from case-patients in their presymptomatic phase exceeds reported transmission rates from other investigations (*1**,**13**,**14*). Possible reasons are the prior evidence that infectiousness peaks around the date of symptom onset, declining thereafter (*15*), and that case-patients probably reduced social contacts themselves once they experienced symptoms or when ordered to self-isolate. A large proportion of cases with presymptomatic transmission in our cluster is further supported by the median serial interval of 3 days.

Of note are the consequences for public health management: first, the need for early detection of COVID-19 cases and for initiation of contact tracing as soon as possible to quarantine close contacts, particularly because short serial intervals may lead to further transmission chains. Second, suspect case-patients or persons with any respiratory illness should immediately self-isolate and inform their contacts met in the presymptomatic and symptomatic phases.

A limitation of our study is that evidence was obtained from a single outbreak and might not be applicable to other settings. We used only information as recalled by the case-patients, which is imperfect and may introduce errors or bias. Because we used development of respiratory symptoms as a proxy for possible SARS-CoV-2 infections among contacts, and because incidence of respiratory illnesses was still high in this winter timeframe, SAR_res_ may be overestimated. However, this possible source of misclassification should be nondifferential between groups. We excluded many HCs because of uncertainties about the potential simultaneous introduction of SARS-CoV-2 in the household, which may have led to an underestimation of SAR among HCs. In the transmission tree, we had to omit various source case–infectee pairs because case-patients’ recalled symptom onset differed substantially from surveillance data and was not plausible (Appendix). Finally, although community transmission of SARS-CoV-2 was deemed unlikely in the affected district at the time, we cannot rule out that some cases acquired infections from other sources.

In conclusion, our study suggests that asymptomatic cases are unlikely to contribute substantially to the spread of SARS-CoV-2. COVID-19 cases should be detected and managed early to quarantine close contacts immediately and prevent presymptomatic transmissions.

Appendix Additional information for asymptomatic and presymptomatic transmission of SARS-CoV-2 in COVID-19 cluster, Germany, 2020.
