# Supplementary material for: Analysis of Asymptomatic and Presymptomatic Transmission in SARS-CoV-2 Outbreak, Germany, 2020
Source: Emerg Infect Dis. 2021 Apr;27(4):1159–63. doi: 10.3201/eid2704.204576 (PMC8007320; doi:10.3201/eid2704.204576)
Supplement: Appendix — Additional information for asymptomatic and presymptomatic transmission of SARS-CoV-2 in COVID-19 cluster, Germany, 2020. [file 20-4576-Techapp-s1.pdf]

Article DOI: https://doi.org/10.3201/eid2704.204576

Analysis of Asymptomatic and Presymptomatic Transmission in SARS-CoV-2 Outbreak, Germany, 2020

Appendix

Methods

Cluster Definition

Our cluster consisted of all severe acute respiratory syndrome coronavirus 2 (SARS-CoV-2) laboratory-confirmed cases living in one district, who were identified by contact tracing activities of the local public health authority (LPHA) following notification of the index case.

Case Survey

Using a comprehensive and standardized questionnaire, a team of scientists from the Robert Koch Institute interviewed all cases belonging to the cluster. We conducted the phone interviews ≈6 weeks after the last date of symptom onset of any of the cases to ascertain correct classification of asymptomatic and symptomatic clinical presentation.

Information on Possible Exposure

With respect to SARS-CoV-2 exposure, we asked each case to provide information regarding possible source cases for their SARS-CoV-2 infection (i.e., case-patients by whom they suspected they had been infected), about their travel history (outside their home district ≤14 days before infection) and whether and which of the district's carnival events they had attended during February 23–26, 2020 (carnival season).

Definition of Asymptomatic and Symptomatic Cases

We defined a case with laboratory-confirmed SARS-CoV-2 infection as symptomatic if he or she recalled at least one of the following symptoms in the 14 days after the last close contact with a case: cough, sore throat, common cold (blocked/runny nose), fever (38.5°C or higher), chills, shortness of breath, pneumonia, headache, back pain, muscle pain, joint pain, loss

of appetite/weight, nausea, vomiting, diarrhea, conjunctivitis, rash, swollen lymph nodes, fatigue, anosmia (loss of smell), or ageusia (loss of taste).

Definition of the Symptom Onset and the Infectious Period

If cases were symptomatic, we defined symptom onset as the first day on which any of the above-listed symptoms had occurred; we did neither document which symptoms were present on the day of symptom onset and which were not, nor did we ask when a specific symptom started or how long it lasted. For cases displaying flu-like symptoms on a day before their most probable exposure, we defined an alternative date of symptom onset as the first date of contact to their most probable source case or most likely exposure (e.g., carnival events). The rationale for this correction was that the influenza season was still not over and no community transmission of SARS-CoV-2 was reported in the district until the carnival weekend.

Based on the reported or assumed (alternative) date of symptom onset of the cases of the cluster, we defined the infectious period for symptomatic cases from 2 days before until 10 days after the date of symptom onset, in accordance with RKI guidelines (https://www.rki.de/DE/Content/InfAZ/N/Neuartiges_Coronavirus/Kontaktperson/Management.html). For asymptomatic cases we assumed that the infectious period started 2 days after and ended 14 days after the presumed infectious encounter with their source case. In the event that there was contact to the source case on several days, we defined the first contact day as the day of the infectious encounter if the source case was a household member and the median date between first and last contact day if the source case was not a household member.

Transmission Tree and Plausibility Checks

We constructed all plausible transmission trees of the cluster based on the cases' information on their possible source cases. To do so, we applied plausibility checks; source case–infectee pairs were regarded implausible when it was not possible to identify a directionality, e.g., if dates of symptom onset and possible exposures were on the same day or interaction took place outside the estimated infectious period.

Determination of Serial Interval, Generation Time and Incubation Period

For each transmission tree, we obtained an empirical distribution of the serial interval (time from symptom onset of the source case to symptom onset in their infectee) based on the date of symptom onset in all resulting source case–infectee pairs. This distribution was then

averaged over all possible trees. Furthermore, because possible exposures were specified as time periods, we used the double-interval-censored approach developed by Reich et al. (*1*) to estimate both the generation time (time from infection of the source case to infection of its infectee) distribution and the incubation period (time from infection to symptom onset) distribution for each tree as Weibull distribution. The estimated distributions for each tree were then combined as follows: for each tree, we sampled 100 parameter instances from the asymptotic multivariate normal distribution for the 2 Weibull parameters. For each parameter combination, we drew 1 instance from the Weibull distribution. The resulting sample of 100 × no. of trees values then constitutes a sample of the generation time distribution and incubation period distribution, which takes both estimation uncertainty and uncertainty of who infected whom and when into account.

Cohort Study among Contact Persons

The study population of the cohort study consisted of all household (HC) and close non-household or other contact persons (OC) whom the cases of our cluster could recall in the interviews and who met the inclusion criteria. We defined a close contact person as a person with >15 minutes face-to-face contact within a maximum distance of 2 m to the case. We did not conduct interviews with the cohort of HC and OC directly.

Inclusion criteria for our cohort analysis were the following: first, we included only HC and OC of those cases, for which it was clear who the primary case in households or other group settings was. Second, we included HC and OC of only those cases in which there was no ambiguity regarding the date of symptom onset ($\leq$2 days difference to the case information in the German surveillance system) (*2*). Third, we included only those HC and OC for whom the interviewed case could provide information about the contact period, if a SARS-CoV-2 test result was available, and if the HC/OC experienced respiratory symptoms after contact with them.

We calculated relative risks (RR) of OC/HC of symptomatic cases for whom the phase could not be specified or with contact in both phases; OC of symptomatic cases with contact only in the presymptomatic phase; and OC of symptomatic cases with contact only in the symptomatic phase by using OC/HC of asymptomatic cases as reference. For this, we used exact Poisson regression and considered $p < 0.05$ as statistically significant.

Software

We entered data from the questionnaires into a database using EpiData version 3.1 (EpiData Association, http://www.epidata.dk). The subsequent analyses were performed with STATA version 15.0 (StataCorp, https://www.stata.com) and R version 3.6.1 (R Foundation for Statistical Computing, https://www.R-project.org).

Ethics

This outbreak investigation was conducted as part of the authoritative official tasks of the LPHA of the district supported by the RKI upon official request in accordance to §4 of the German Protection against Infection Act. Therefore, this investigation was exempt from institutional review.

Results

Cohort Study

Among laboratory-confirmed OC, most (75%; 15/20) had contact with their source case only during the presymptomatic period of the case; 10% (2/20) of laboratory-confirmed OC had contact with their source case only in the symptomatic period of the case. For 15% (3/20), contact could not be specified by the symptomatic case, in which phase of the infectious period the contact took place, or contact took place in both phases.

Among OC with respiratory symptoms, the proportion that had contact only in the presymptomatic period of their source case was similar (76%; 22/29). Furthermore, 3% (1/29) of OC experienced symptoms after contact with the source case only in their symptomatic period and 14% (4/29) if the symptomatic period could not be specified or if contact occurred in both phases. In this study, 7% (2/29) of OC from asymptomatic cases later became symptomatic.

References

1. Reich NG, Lessler J, Cummings DA, Brookmeyer R. Estimating incubation period distributions with coarse data. Stat Med. 2009;28:2769–84. PubMed https://doi.org/10.1002/sim.3659

2. Faensen D, Claus H, Benzler J, Ammon A, Pfoch T, Breuer T, et al. SurvNet@RKI—a multistate electronic reporting system for communicable diseases. Euro Surveill. 2006;11:100–3. PubMed https://doi.org/10.2807/esm.11.04.00614-en

Appendix Table. Distributions of the calculated serial interval, incubation period and generation time as inferred from the 53 COVID-19 cases of the cluster in a district in Southern Germany.

	Quantiles							
Time	1%	5%	25%	50%	75%	95%	99%	Mean
Serial interval, d	−2.0	−1.0	1.0	3.0	6.0	15.0	22.0	4.5
Generation time, d	0.1	0.3	1.7	3.6	6.6	13.1	21.6	4.9
Incubation period, d	0.3	0.8	2.5	4.3	6.5	10.6	14.3	4.8

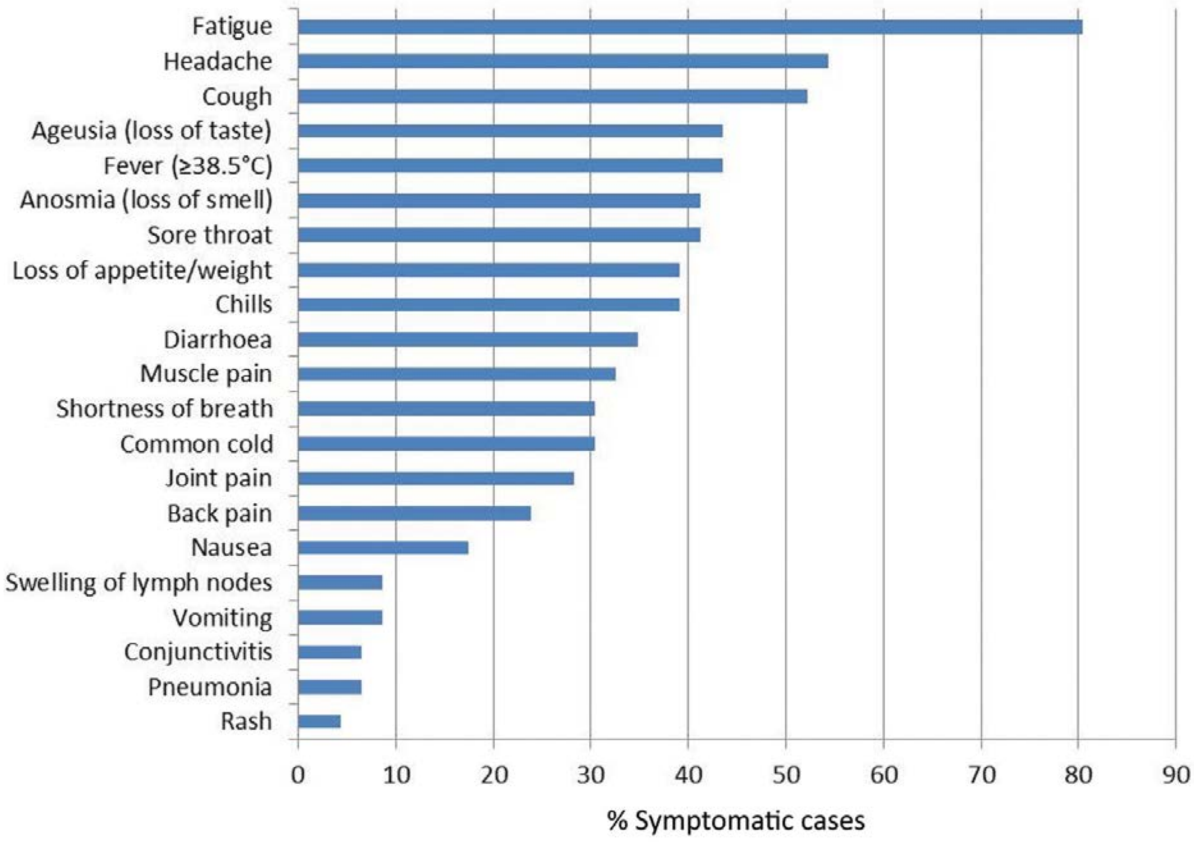

Appendix Figure 1. Reported symptoms among symptomatic cases from the COVID-19 cluster in a district in Southern Germany (n = 46).

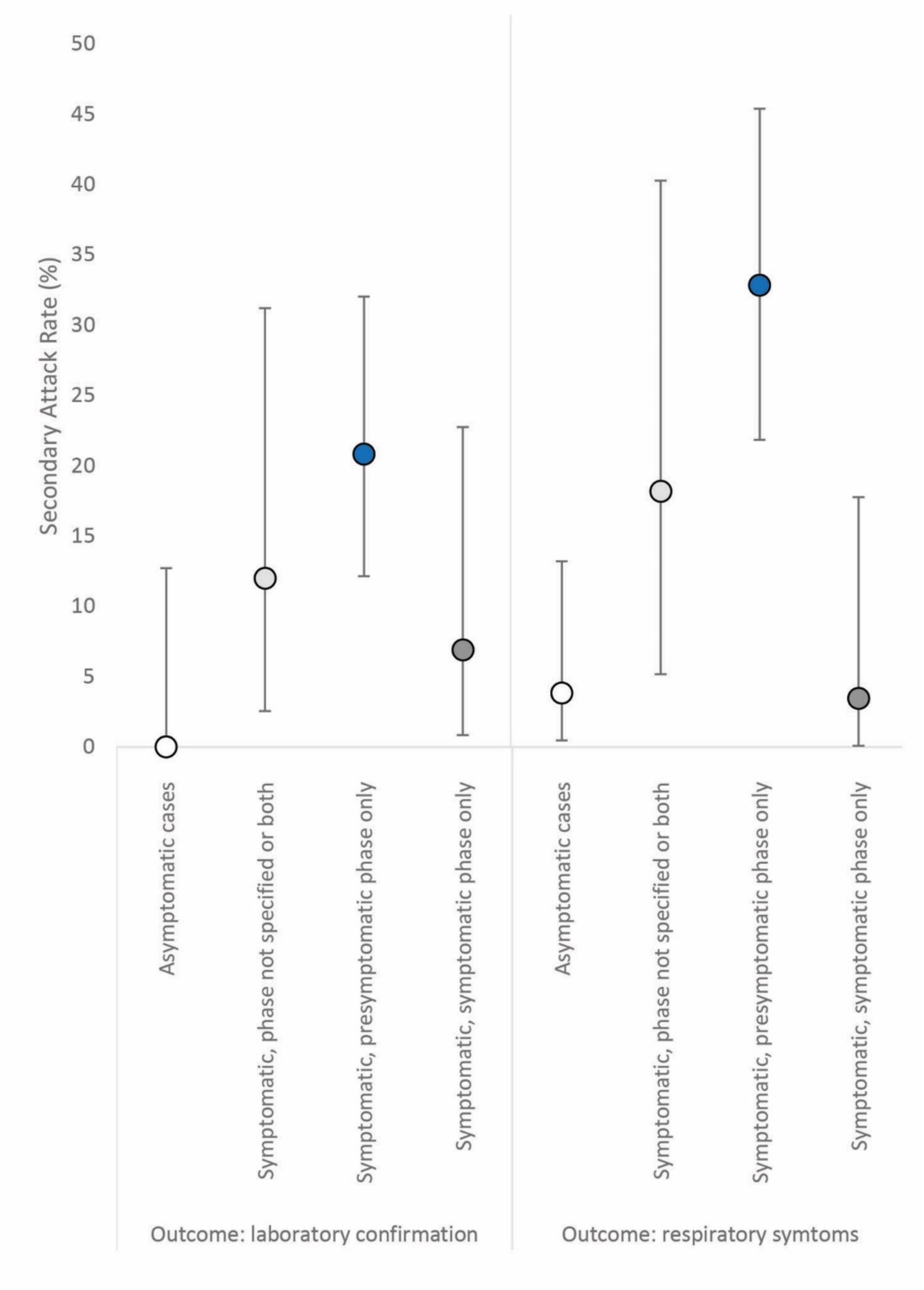

Appendix Figure 2. Secondary attack rates (SAR) among non-household/other contacts in the COVID-19 cluster in a district in southern Germany. SAR for the outcomes of laboratory-confirmed COVID-19 and the development of respiratory symptoms in contacts of cases belonging to the cluster are shown for each group of the infectious phase/clinical presentation of the case. Bars represent 95% CI. White dot represents SAR among contacts from asymptomatic cases; light gray, SAR among contacts from symptomatic cases where phase was not specified or contact in both phases; blue, SAR among contacts from symptomatic cases with contact in presymptomatic phase only; dark grey, SAR among contacts from symptomatic cases with contact in symptomatic phase only.
